# A retrospective study of disproportionality analysis signals between proprotein convertase subtilisin/kexin 9 inhibitor use and respiratory, thoracic, and mediastinal disorders

**DOI:** 10.1097/MD.0000000000050089

**Published:** 2026-08-28

**Authors:** Hongzhu Chen, Lijing Li

**Affiliations:** a School of Pharmaceutical Sciences, Jilin University, Changchun, Jilin Province, P.R. China; b Department of Pharmacy, Changchun University of Chinese Medicine, Changchun, Jilin Province, P.R. China.

**Keywords:** adverse drug reaction, FAERS, PCSK9 inhibitors, respiratory, thoracic, and mediastinal diseases

## Abstract

This study aims to investigate emerging signals and clinical characteristics associated with adverse event reports of respiratory, thoracic, and mediastinal diseases (RTMD) related to proprotein convertase subtilisin/kexin 9 inhibitors (PCSK9i) in the FDA Adverse Event Reporting System (FAERS) database. PCSK9i-related RTMD adverse event reports were extracted from the FAERS database (covering the period from the first quarter of 2016 to the fourth quarter of 2023), and the clinical characteristics and onset times of these reports were analyzed. The reporting odds ratio (ROR) and the Bayesian Confidence Propagation Neural Network (BCPNN) were used to calculate disproportionate signals in the adverse event reports. A total of 17,063 PCSK9i-related RTMD adverse event reports were extracted from the FAERS database. A summary of these reports revealed that the median age of the reported population was 68 years (interquartile range: 61–74 years); the proportion of female patients was higher than that of male patients (56.53% vs 40.56%). The United States submitted the highest number of adverse event reports (n = 15,031, 88.09%), followed by Germany (n = 1076, 6.31%). The median time to onset for adverse event reports was 7.5 days (interquartile range = 0.5–77.5 days); the median time to onset for reports related to alirocumab was 14.5 days (interquartile range = 1.5–100.5 days), while for evolocumab it was only 6.5 days (interquartile range = 0.5–70.5 days). Disproportionate analysis of adverse event reports revealed signals for both evolocumab (ROR = 1.18, 95% CI: 1.16–1.20; information component [IC] = 0.35, IC_025_ = 0.33) and alirocumab (ROR = 1.50, 95% CI: 1.45–1.55; IC = 0.41, IC_025_ = 0.38). Among these, rhinorrhea, cough, oropharyngeal pain, nasal congestion, and dysphonia were the most frequently reported preferred terms (PTs). The results of this study indicate the presence of a disproportionate signal of RTMD adverse events associated with PCSK9i in the FAERS database; however, this merely suggests that such events warrant further attention in reports related to PCSK9i and does not establish a causal relationship between the 2.

## 1. Introduction

Proprotein convertase subtilisin/kexin 9 inhibitors (PCSK9i) are a new class of lipid-lowering drugs used to treat patients with familial hyperlipidemia and those at high cardiovascular risk.^[1]^ In 2015, the US Food and Drug Administration (FDA) and the European Medicines Agency (EMA) approved 2 PCSK9i, alirocumab and evolocumab.^[2]^ In 2019, the European Society of Cardiology (ESC)/European Atherosclerosis Society (EAS) guidelines recommended PCSK9i for the treatment of dyslipidemia.^[3]^ Recent clinical trials have shown that adding PCSK9i to statin therapy can further lower low-density lipoprotein cholesterol (LDL-C) levels and reduce the risk of atherosclerotic cardiovascular disease (ASCVD).^[4-6]^

Although PCSK9i offer substantial clinical benefits, findings from multiple studies regarding PCSK9i related adverse events remain inconsistent. An analysis based on hospital registry systems and pharmacovigilance databases suggests that the most common adverse reactions associated with PCSK9i are upper respiratory tract infections, nasopharyngitis, and myalgia.^[2]^ Results from multiple randomized, double-blind trials show that the risk of respiratory infection-related adverse events in the PCSK9i treatment group was significantly higher than in the placebo group, particularly for influenza-like symptoms and nasopharyngitis.^[7-10]^ However, other studies have reported similar rates of respiratory-related adverse events between the PCSK9i and placebo groups, with the only difference being in the incidence of injection site pain.^[11,12]^

The FDA Adverse Event Reporting System (FAERS) is a publicly available database that collects adverse event reports submitted by healthcare professionals, consumers, and pharmaceutical manufacturers worldwide.^[13]^ The association between PCSK9i and respiratory adverse events remains controversial; therefore, this study is a retrospective analysis based on the FAERS database aimed at exploring disproportionate signals and clinical characteristics of respiratory, thoracic, and mediastinal disorders (RTMDs) adverse event reports associated with PCSK9i.

## 2. Materials and methods

### 2.1. Data source

This study is a retrospective analysis using the FAERS database aimed at identifying signals of disproportionate adverse event reporting and clinical characteristics associated with PCSK9i. The database contains information across 7 dimensions: drug information (DRUG), treatment start and end dates (THER) of reported drugs, adverse event terminology (REAC), reporting source (RPSR), patient demographics (DEMO), outcomes (OUTC), and indications for use (INDI). This retrospective analysis included all reports submitted from the first quarter of 2016 to the fourth quarter of 2023.

### 2.2. Adverse event identification

Adverse events were identified using preferred terms (PTs) from the Medical Dictionary for Regulatory Activities (MedDRA; Version 24.0). MedDRA has a multilevel structure, and PTs provide detailed descriptions of individual medical concepts (such as symptoms and disease diagnoses). Different PTs are classified into broader system organ classes (SOCs) based on etiology, site of occurrence, or purpose. In this study, adverse event reports were extracted from the “Respiratory, Thoracic and Mediastinal Disorders” (SOC: 10038738; RTMD) category in MedDRA for subsequent analysis.

### 2.3. Data extraction

In accordance with FDA guidelines^[14]^, duplicate data were removed. When the CASEID was the same, the most recent FDA_DT was selected to ensure that the analysis reflected the most up-to-date results for each case; when both the CASEID and FDA_DT were the same, the higher PRIMARYID was selected to ensure that the most recent report was chosen.

To retrieve PCSK9i-related adverse event reports from FAERS, this study used the FDA-approved generic and brand names of PCSK9i, to search for and identify reports in the DRUG file (alirocumab, praluent, evolocumab, and repatha). To improve the accuracy of the results, only reports classified as “primary suspected drug” in the ROLE_COD field were included.

### 2.4. Signal mining

We used the reporting odds ratio (ROR)^[15]^ and the Bayesian confidence propagation neural network (BCPNN) algorithm^[16]^ to investigate whether there was a signal in the adverse event reports related to PCSK9i in the RTMD. The calculation formulas and criteria for both algorithms are detailed in Table S1, Supplemental Digital Content 1; results that met the criteria of both algorithms were considered to indicate a signal.

Descriptive analysis was used to summarize the clinical characteristics of patients in the reports. The Weibull distribution was employed to model the temporal trends in time to onset, with the shape parameter (β) used to characterize the time-to-onset patterns: β < 1 indicates an early failure type, where the frequency of onset decreases over time; β = 1 indicates a random failure type, where the frequency of adverse event reports remains constant over time; β > 1 indicates a wear-out failure type, where the frequency of adverse event reports increases over time. Data analysis and statistical calculations were performed using R software (version 4.3.3; The R Foundation for Statistical Computing).

### 2.5. Ethical considerations

This study used de-identified data from the publicly available FAERS database and was exempt from Institutional Review Board approval.

## 3. Result

### 3.1. Number of RTMD adverse events

Between the first quarter of 2016 and the fourth quarter of 2023, the FAERS database received 304,168 adverse event reports for PCSK9i, with RTMD adverse events accounting for 17,063 of these (5.61% of all adverse events). The proportion of RTMD reports among all adverse event reports varied slightly between the 2 PCSK9i agents: 3622 RTMD adverse event reports involving alirocumab accounted for 6.73% of all adverse events, while 13,441 RTMD adverse event reports involving evolocumab accounted for 5.37% of all adverse events (Table 1).

**Table 1:** The number of PCSK9i-associated RTMD adverse event reports in the FAERS database from Q1 2016 to Q4 2023.

	With RTMD AE cases	Without RTMD AE cases	All AE cases	RTMD AEs/all AEs (%)
Overall	17,063	2,87,105	3,04,168	5.61%
Evolocumab	13,441	2,36,921	2,50,362	5.37%
Alirocumab	3622	50,184	53,806	6.73%

AE = adverse event, FAERS = FDA Adverse Event Reporting System, PCSK9i = proprotein convertase subtilisin/kexin 9 inhibitor, RTMD = respiratory, thoracic, and mediastinal disorders.

### 3.2. Clinical characteristics of RTMD adverse events

The clinical features of PCSK9i-related adverse event reports are shown in Table 2. Compared to male patients (n = 6921, 40.56%), the percentage of female patients (n = 9646, 56.53%) was substantially greater. The median age of patients treated with evolocumab was 68 years (IQR = 61–74) among the 10,053 adverse event reports that included age information. The median age of patients treated with alirocumab was also 68 years (IQR = 62–74). Germany (n = 1076, 6.31%) and the United States (n = 15,031, 88.09%) provided the most adverse event reports.

**Table 2:** Characteristics of PCSK9i-associated respiratory, thoracic, and mediastinal disorders adverse event reports from the FAERS database (Q1 2016–Q4 2023).

Characteristics	Evolocumab (n = 13441)		Alirocumab (n = 3622)		PCSK9i (n = 17063)	
Demographics						
Reporter gender	%	n	%	n	%	n
Women	59.17%	7953	46.74%	1693	56.53%	9646
Men	40.20%	5403	41.91%	1518	40.56%	6921
Unknown	0.63%	445	11.35%	411	2.91%	496
Age						
Median (IQR)	68 (61–74)		68 (62–74)		68 (61–74)	
Unknown	25.21%	3388	36.89%	1336	27.69%	4724
Reporting characteristics						
Reporter region	%	n	%	n	%	n
United States	91.07%	12,241	77.03%	2790	88.09%	15,031
Germany	4.06%	546	14.63%	530	6.31%	1076
Canada	1.50%	201	1.30%	47	1.45%	248
Reporting year	%	n	%	n	%	n
2016	7.77%	1045	10.55%	382	8.36%	1427
2017	8.01%	1077	15.18%	550	9.54%	1627
2018	55.79%	7499	18.14%	657	47.80%	8156
2019	7.53%	1012	21.54%	780	10.50%	1792
2020	4.62%	621	8.50%	308	5.44%	929
2021	3.94%	530	4.58%	166	4.08%	696
2022	5.54%	744	11.32%	410	6.76%	1154
2023	6.79%	913	10.19%	369	7.51%	1282
Occupation of reporter	%	n	%	n	%	n
Health professional	0.59%	7905	0.26%	955	0.52%	8860
Non health professional	0.40%	5443	0.73%	2660	0.47%	8103
Indication	%	n	%	n	%	n
Hypercholesterolaemia	24.99%	3359	13.20%	478	22.49%	3837
Blood cholesterol increased	17.12%	2301	11.13%	403	15.85%	2704
Hyperlipidaemia	8.83%	1187	13.42%	486	9.80%	1673
Type iia hyperlipidemia	5.54%	744	9.22%	334	6.32%	1078
Outcome	%	n	%	n	%	n
Other serious outcomes	19.22%	2584	26.67%	966	20.81%	3550
Hospitalization	6.33%	851	8.97%	325	6.89%	1176
Life threatening	0.90%	121	0.58%	21	0.83%	142
Death	0.67%	90	1.55%	56	0.86%	146
Disability	0.62%	84	1.08%	39	0.72%	123

FAERS = FDA Adverse Event Reporting System, PCSK9i = proprotein convertase subtilisin/kexin 9 inhibitor.

The most adverse event reports were received in 2018 (n = 8156), with a notably high number of complaints involving evolocumab (n = 7499). The number of adverse event reports fell dramatically in 2020 (n = 929) and 2021 (n = 696), before rebounding in 2022 (n = 1154). The majority of reports were submitted by physicians (n = 7254, 42.51%) and consumers (n = 6664, 39.06%). According to the collected data, the most often reported indications were hypercholesterolemia (n = 3837, 22.49%) and high blood cholesterol (n = 2704, 15.85%). The most common adverse event outcomes were other serious adverse events (n = 3550, 20.81%) and hospitalization (n = 1176, 6.89%).

### 3.3. Time to onset analysis

The time-to-onset (TTO) and Weibull distribution analyses included 9902 of the 17,063 RTMD reports with reliable time information (6905 for evolocumab and 2997 for alirocumab). The median time to onset of all RTMD adverse events for PCSK9i was 7.5 days (IQR = 0.5–77.5 days), with 63.55% of adverse events occurring during the first 30 days. Among the individual PCSK9i, alirocumab had a greater median time to onset of 14.5 days (IQR = 1.5–100.5 days), while evolocumab had a shorter median time to onset of 6.5 days.

Table 3 shows the detailed parameters from the Weibull distribution study results. Weibull distribution analysis showed that both evolocumab (shape parameter β = 0.62, 95% CI: 0.61–0.62) and alirocumab (shape parameter β = 0.62, 95% CI: 0.60–0.63) had an early failure style, indicating that adverse event reporting was concentrated in the short term after treatment initiation.

**Table 3:** Time to onset of respiratory, thoracic, and mediastinal disorders adverse events and Weibull distribution analysis.

Drug	TTO (d)			Scale parameter		Shape parameter		Failure type
	n	Median (IQR)	Min–max	α	95% CI	β	95% CI	
Evolocumab	6905	6.5	0.5–70.5	167.57	160.80–174.33	0.62	0.61–0.62	Early failure
Alirocumab	2997	14.5	1.5–100.5	131.43	123.37–139.49	0.62	0.60–0.63	Early failure

CI = confidence interval, IQR = interquartile range, TTO = time-to-onset.

### 3.4. The disproportionate reporting signal of RTMD adverse events

We examined the categories and number of adverse event reports related to PCSK9i. In the evolocumab group, the top 5 most frequently reported adverse events by number of reports were: rhinorrhea (n = 3,151, 23.44%), cough (n = 1737, 12.92%), dyspnea (n = 1691, 12.58%), oropharyngeal pain (n = 1283, 9.55%), and nasal congestion (n = 563, 4.19%). In the alirocumab group, the top 5 PTs were cough (n = 586, 16.18%), dyspnea (n = 541, 14.94%), rhinorrhea (n = 491, 13.56%), oropharyngeal discomfort (n = 270, 7.45%), and nasal congestion (n = 180, 4.97%).

The disproportionate signal values for SOC of PCSK9i-related RTMDs were calculated against the context of the full FAERS database. Table 4 shows the results of evolocumab-related RTMD adverse events: ROR = 1.18, 95% CI: 1.16–1.20; information component (IC) = 0.35, IC_025_ = 0.33. Alirocumab-related RTMD adverse events: ROR = 1.50, 95% CI: 1.45–1.55; IC = 0.41; IC_025_ = 0.38. Both showed a disproportionate signal, with evolocumab-related RTMD adverse events being weaker.

**Table 4:** Signal detection for respiratory, thoracic, and mediastinal disorders adverse events of PCSK9i in the FAERS database from Q1 2016 to Q4 2023 (SOC level).

SOC	Evolocumab		Alirocumab	
	ROR (95% CI)	IC (IC_025_)	ROR (95% CI)	IC (IC_025_)
RTMD	1.18 (1.16–1.2)	0.35 (0.33)	1.5 (1.45–1.55)	0.41 (0.38)

CI = confidence interval, FAERS = FDA Adverse Event Reporting System, IC = information component, PCSK9i = proprotein convertase subtilisin/kexin 9 inhibitor, PT = preferred term, ROR = Reported odds ratios, RTMD = Respiratory, thoracic, and mediastinal disorders, SOC = system organ class.

Next, we conducted a disproportionate analysis on PTs with ≥10 reported cases in the RTMD SOC. Table 5 shows the overall findings from the disproportionate analysis. After screening using positive signal criteria, we discovered PTs with a disproportionate signal and a large number of reported cases: rhinorrhea (evolocumab: n = 3151, ROR = 12.47, IC_025_ = 3.45; alirocumab: n = 491, ROR = 8.37, IC_025_ = 2.9), cough (evolocumab: n = 1737, ROR = 1.49, IC_025_ = 0.49; alirocumab: n = 586, ROR = 2.34, IC_025_ = 1.10), oropharyngeal pain (evolocumab: n = 1283, ROR = 3.42, IC_025_ = 1.66; alirocumab: n = 270, ROR = 3.3, IC_025_ = 1.53), nasal congestion (evolocumab: n = 563, ROR = 2.44, IC_025_ = 1.15; alirocumab: n = 180, ROR = 3.61, IC_025_ = 1.63), and dysphonia (evolocumab: n = 482, ROR = 2.05, IC_025_ = 0.89; alirocumab: n = 164, ROR = 3.24, IC_025_ = 1.46; Table 5). Notably, several PTs with lower reporting frequencies showed higher disproportionality signals than more commonly reported PTs. For example, alirocumab revealed a significant signal for obstructive sleep apnea syndrome (n = 48, ROR = 54.14, IC_025_ = 5.20), while evolocumab showed a strong signal for paranasal sinus hypersecretion (n = 119, ROR = 9.20, IC_025_ = 2.84). The findings show that there may be adverse events with low incidence but strong signals. Nonetheless, these findings should be interpreted with caution, as disproportionality signals generated from a limited number of instances are prone to reporting fluctuations, coding variances, and residual confounding factors.

**Table 5:** Signal detection for respiratory, thoracic, and mediastinal disorders adverse events of PCSK9i in the FAERS database from Q1 2016 to Q4 2023 (PT level).

PT	Evolocumab (N)	ROR	IC_025_	PT	Alirocumab (N)	ROR	IC_025_
**Rhinorrhoea**	**3151**	**12.47**	**3.45**	**Cough**	**586**	**2.34**	**1.10**
**Cough**	**1737**	**1.49**	**0.49**	**Dyspnoea**	**541**	**1.12**	**0.03**
Dyspnoea	1691	0.75	−0.49	**Rhinorrhoea**	**491**	**8.37**	**2.9**
**Oropharyngeal pain**	**1283**	**3.42**	**1.66**	**Oropharyngeal pain**	**270**	**3.30**	**1.53**
**Nasal congestion**	**563**	**2.44**	**1.15**	**Nasal congestion**	**180**	**3.61**	**1.63**
**Dysphonia**	**482**	**2.05**	**0.89**	**Dysphonia**	**164**	**3.24**	**1.46**
**Sinus disorder**	**339**	**4.18**	**1.87**	**Wheezing**	**107**	**2.1**	**0.78**
**Sneezing**	**277**	**3.15**	**1.46**	**Sinus disorder**	**87**	**4.91**	**1.97**
**Throat irritation**	**274**	**1.6**	**0.49**	**Dyspnoea exertional**	**86**	**2.47**	**0.99**
Productive cough	226	1.11	−0.04	**Sneezing**	**76**	**3.98**	**1.65**
**Respiratory tract congestion**	**211**	**3.73**	**1.67**	**Obstructive airways disorder**	**72**	**6.43**	**2.33**
Epistaxis	195	0.63	−0.87	**Throat irritation**	**70**	**1.89**	**0.57**
Wheezing	171	0.72	−0.7	Epistaxis	54	0.81	−0.69
Asthma	167	0.37	−1.65	Productive cough	51	1.17	−0.17
**Upper-airway cough syndrome**	**162**	**4.47**	**1.89**	**Obstructive sleep apnoea syndrome**	**48**	**54.14**	**5.2**
Dyspnoea exertional	159	0.98	−0.26	**Pneumothorax**	**47**	**3.38**	**1.33**
**Throat tightness**	**136**	**1.35**	**0.19**	**Sinus congestion**	**44**	**4.27**	**1.65**
**Sinus congestion**	**132**	**2.77**	**1.2**	Asthma	36	0.37	−1.89
Respiratory disorder	126	1.12	−0.1	**Respiratory tract congestion**	**33**	**2.66**	**0.91**
**Paranasal sinus hypersecretion**	**119**	**9.2**	**2.84**	**Throat tightness**	**31**	**1.43**	**0.01**
**Aphonia**	**110**	**1.95**	**0.68**	**Upper-airway cough syndrome**	**27**	**3.38**	**1.21**
**Dry throat**	**93**	**2.35**	**0.92**	Sleep apnoea syndrome	25	1.42	−0.07
Chronic obstructive pulmonary disease	88	0.44	−1.49	Chronic obstructive pulmonary disease	23	0.54	−1.49
**Pharyngeal oedema**	**77**	**1.74**	**0.46**	**Pharyngeal oedema**	**22**	**2.3**	**0.6**
Lung disorder	66	0.34	−1.91	**Oropharyngeal discomfort**	**21**	**2.5**	**0.7**
**Pulmonary congestion**	**63**	**1.38**	**0.1**	**Nasal disorder**	**16**	**6.21**	**1.92**
Obstructive airways disorder	63	1.2	−0.1	Respiratory disorder	16	0.66	−1.3
Pharyngeal swelling	54	1.07	−0.29	Aphonia	15	1.23	−0.42
Pulmonary embolism	50	0.16	−2.99	**Paranasal sinus discomfort**	**15**	**3.94**	**1.25**
Sleep apnoea syndrome	49	0.59	−1.16	Pharyngeal swelling	15	1.38	−0.25
Pneumothorax	46	0.71	−0.92	**Paranasal sinus hypersecretion**	**14**	**4.76**	**1.49**
**Obstructive sleep apnoea syndrome**	**45**	**10.77**	**2.88**	Dry throat	14	1.63	−0.04
Oropharyngeal discomfort	44	1.12	−0.27	**Nasal discomfort**	**14**	**2.14**	**0.35**
Choking	39	0.5	−1.45	Pleural effusion	13	0.27	−2.67
Pleural effusion	36	0.16	−3.12	Lung disorder	13	0.31	−2.45
Pulmonary oedema	34	0.2	−2.79	Respiratory distress	13	0.6	−1.51
**Respiratory symptom**	**31**	**2.35**	**0.7**	Pulmonary congestion	12	1.22	−0.51
Sputum discoloured	31	0.71	−1.01	**Sinus pain**	**11**	**3.94**	**1.14**
Nasal dryness	31	1.24	−0.21	Pulmonary oedema	11	0.31	−2.54
Respiration abnormal	29	0.86	−0.74	Pulmonary embolism	11	0.17	−3.39
Nasal discomfort	29	0.95	−0.6	Choking	11	0.66	−1.44
**Nasal disorder**	**24**	**2**	**0.41**	**Choking sensation**	**10**	**2.27**	**0.31**
Paranasal sinus discomfort	24	1.35	−0.15	**Increased upper airway secretion**	**10**	**5.07**	**1.46**
Throat clearing	23	1.06	−0.51				
Respiratory distress	22	0.22	−2.8				
**Sinus pain**	**22**	**1.7**	**0.15**				
**Pharyngeal disorder**	**21**	**1.93**	**0.31**				
**Upper respiratory tract congestion**	**19**	**1.85**	**0.22**				
Interstitial lung disease	19	0.1	−4.01				
Respiratory failure	19	0.07	−4.47				
Haemoptysis	18	0.16	−3.3				
**Increased upper airway secretion**	**18**	**1.96**	**0.3**				
Pulmonary mass	17	0.25	−2.67				
Choking sensation	16	0.78	−1.06				
Hiccups	16	0.54	−1.6				
**Pharyngeal erythema**	**15**	**1.85**	**0.15**				
**Nasal pruritus**	**15**	**2.41**	**0.53**				
Respiratory arrest	15	0.2	−3.02				
Pulmonary pain	13	0.86	−0.98				
Pulmonary thrombosis	13	0.27	−2.67				
Rhinalgia	12	1.16	−0.59				
Pulmonary fibrosis	12	0.17	−3.31				
Emphysema	11	0.32	−2.48				
Lower respiratory tract congestion	11	1.11	−0.69				
Pleurisy	10	0.49	−1.9				

Bold: RTMD adverse events disproportionate positive signal.

FAERS = FDA Adverse Event Reporting System, IC = information component, PCSK9i = proprotein convertase subtilisin/kexin 9 inhibitor, PT = preferred term, ROR = Reported odds ratios, RTMD = Respiratory, thoracic, and mediastinal disorders.

In the context of atorvastatin, rosuvastatin, and ezetimibe, disproportionate signals were detected for the SOC of alirocumab-related RTMD. When compared to the background of atorvastatin and ezetimibe, evolocumab showed a disproportionate risk signal, but no positive signal was seen when compared to the background of rosuvastatin (Table 6).

**Table 6:** Disproportionality analysis comparing PCSK9 inhibitors with other lipid-lowering therapies.

PCSK9i	Comparator	ROR (95% CI)
**Evolocumab**	**Atorvastatin**	**1.04 (1.01–1.07**)
**Alirocumab**	**Atorvastatin**	**1.32 (1.27–1.37**)
Evolocumab	Rosuvastatin	0.92 (0.89–0.96)
**Alirocumab**	**Rosuvastatin**	**1.17 (1.12–1.23**)
**Evolocumab**	**Ezetimibe**	**1.08 (1.02–1.14**)
**Alirocumab**	**Ezetimibe**	**1.37 (1.30–1.45**)

Bold indicates the drug exhibited a disproportionate signal compared with the comparator.

CI = confidence interval, PCSK9i = proprotein convertase subtilisin/kexin 9 inhibitor, ROR = reported odds ratios.

### 3.5. Co-reported adverse events with RTMD adverse events

**Figure 1.**
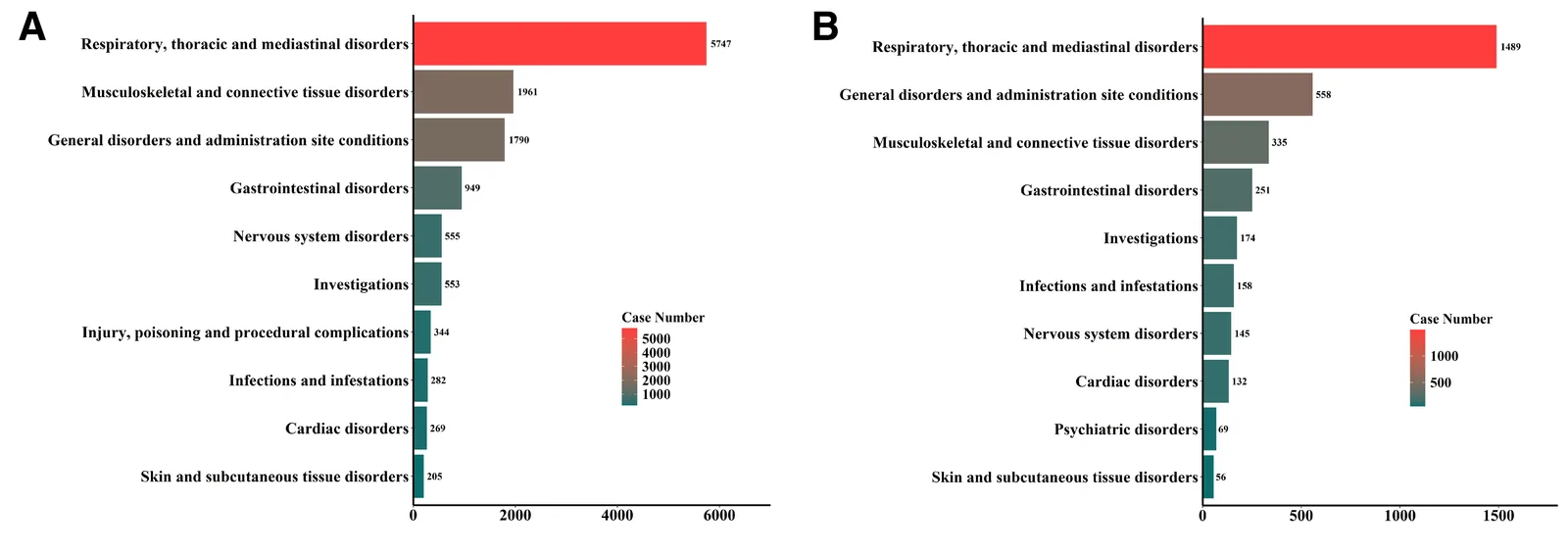
The top 10 overlapping system organ classes (SOCs) were observed between RTMD adverse events and other concurrent adverse events. For evolocumab, the most frequently overlapped SOCs were “musculoskeletal and connective tissue disorders” (n = 1961), “general disorders and administration site conditions” (n = 1790), and “gastrointestinal disorders” (n = 949) (A). For alirocumab, the predominant overlapping SOCs included “general diseases and administration site conditions” (n = 558), “musculoskeletal disorders” (n = 335), and “gastrointestinal disorders” (n = 251) (B). RTMD = respiratory, thoracic, and mediastinal diseases.

There were more combinations of RTMD adverse events and other concomitant adverse events, and Figure 1 depicts the top ten overlapping SOCs reported by patients after PCSK9i treatment. The most common overlapping SOCs for evolocumab included’musculoskeletal and connective tissue disorders’ (n = 1961), ‘general disorders and administration site conditions (n = 1790), and “gastrointestinal disorders” (n = 949). Alirocumab’s most prevalent overlapping SOCs were “general diseases and administration site conditions” (n = 558), “musculoskeletal disorders” (n = 335), and “gastrointestinal disorders” (n = 251).

### 3.6. Age and gender characteristics of RTMD adverse events reports

**Figure 2.**
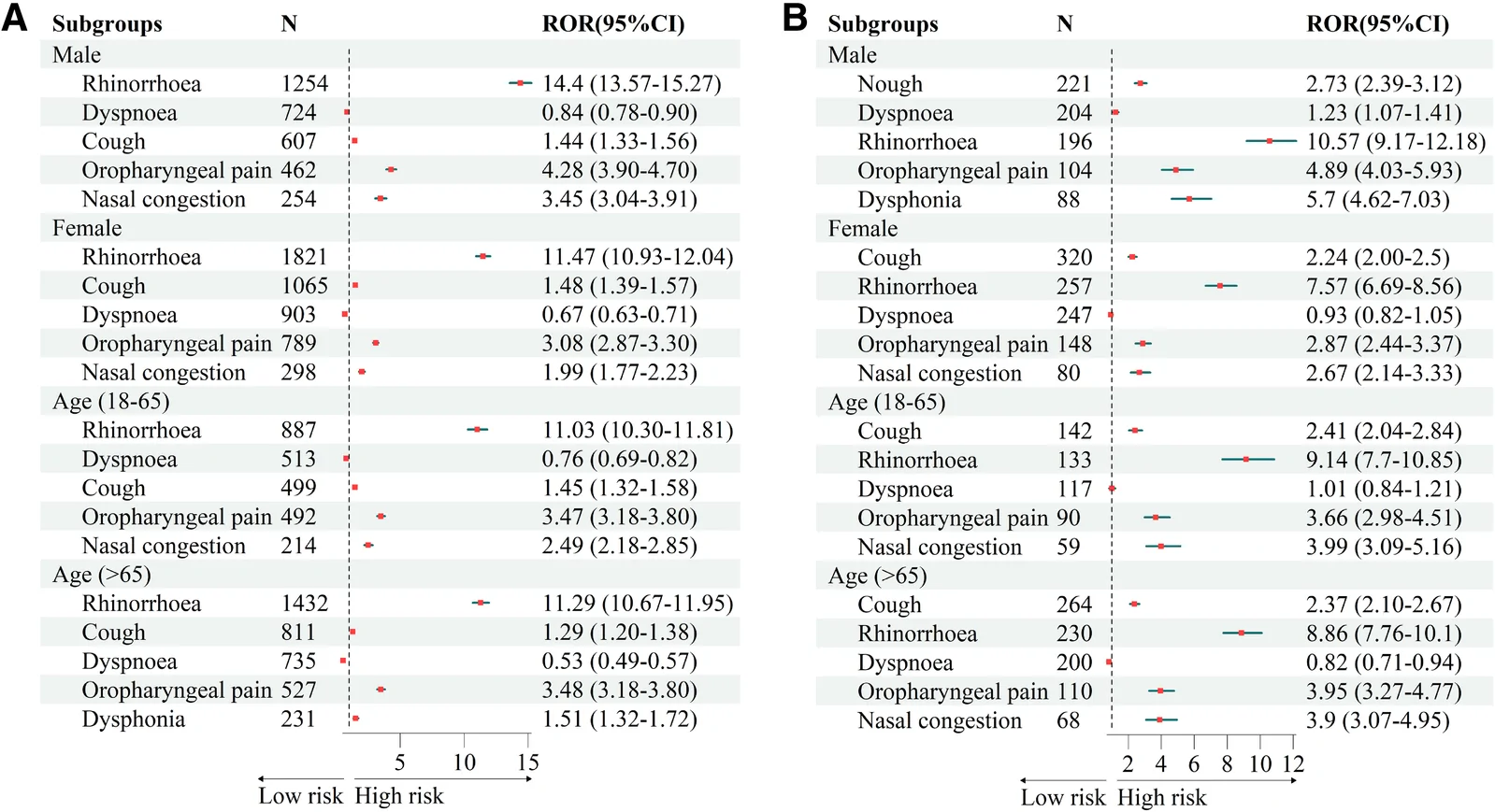
Age and sex subgroup characteristics of PCSK9i-related RTMD adverse events. (A) Evolocumab; (B) alirocumab. CI = confidence interval, PCSK9i = proprotein convertase subtilisin/kexin 9 inhibitor, ROR = reported odds ratios, RTMD = respiratory, thoracic, and mediastinal disorders.

We investigated the age and gender-specific features of PCSK9i-related RTMD adverse events. In evolocumab-related reports, we found that male patients (n = 1,254, ROR = 14.4, 95% CI: 13.57–15.27) and female patients (n = 1,821, ROR = 11.47, 95% CI: 10.93–12.04) most commonly reported rhinorrhea, with women reporting more than males. Rhinorrhea was the most frequently reported condition in both the 18 to 65 and ≥65 age groupings, with 887 reports in the 18 to 65 age group (ROR = 11.03, 95% CI: 10.3–11.81) and 1432 reports in the ≥ 65 age group (ROR = 11.29, 95% CI: 10.67–11.95; Fig. 2).

Figure 2 shows that cough was the most commonly reported adverse event in male patients when using alirocumab (n = 221, ROR = 2.73, 95% CI: 2.39–3.12). Cough was also the most commonly reported adverse event in female patients, with more reports than in males (n = 320, ROR = 2.24, 95% CI: 2.00–2.25). Cough was the most commonly reported occurrence in both the 18–65 and ≥ 65 age subgroups: 142 cases were reported in the 18 to 65 group, with a ROR of 2.41 (95% CI: 2.04–2.84); 264 cases were recorded in the ≥65 age group, with a ROR of 2.37 (95% CI: 2.10–2.67).

## 4. Discussion

In addition to their beneficial effects on lipid metabolism and cardiovascular disease, new research has revealed that PCSK9i are involved in the regulation of glucose metabolism, the neurological system, inflammation, and renal function, among other things.^[17-19]^ Currently, there is a lack of studies that examine the comprehensive aspects of PCSK9i-related respiratory and pulmonary adverse events. Based on the FAERS database, this study extracted PCSK9i-related RTMD adverse event reports, detailed their clinical characteristics, and estimated disproportionate signals.

Early clinical trials found no association between PCSK9i and RTMD adverse events; both the ODYSSEY LONG TERM and OSLER studies revealed good long-term safety for evolocumab and alirocumab.^[12]^ This study found that during the first quarter of 2016 to the fourth quarter of 2023, PCSK9i-related RTMD adverse event reports accounted for 5.61% (17,063/304,168) of all adverse event reports in the FAERS database, which is a comparatively low proportion. Among these, 13,441 reports of RTMD adverse events were related to evolocumab, accounting for 5.37% of all adverse event reports; 3622 reports of RTMD adverse events were related to alirocumab, accounting for 6.73% of all adverse event reports. The signal strength of the disproportionate incidence of RTMD adverse event reports differs between the 2 PCSK9i agents: alirocumab (ROR = 1.50, 95% CI: 1.45–1.55) has a greater signal than evolocumab (ROR = 1.18, 95% CI: 1.16–1.2). At the PT level, among reports involving the 2 PCSK9i drugs, rhinorrhea, cough, oropharyngeal pain, nasal congestion, and dysphonia were the most common adverse events with a signal. Overall, previous randomized controlled trials and package insert data did not indicate a clear safety imbalance in the respiratory system; however, clinical trials did report events such as nasopharyngitis, upper respiratory tract infection, influenza, cough, and oropharyngeal pain, though the absolute differences compared to placebo were generally small.^[7-20]^ The EMC registration study found that 16.2% of patients treated with PCSK9i developed upper respiratory tract infections, with nasopharyngitis being the most common; this was also the most frequently reported adverse event in the VigiLyze and Lareb databases.^[2]^ The FOURIER and ODYSSEY studies also found an increase in the number of nasopharyngitis cases after PCSK9i therapy.^[2]^ These findings are consistent with the patterns found in the current trial, indicating that this type of adverse event requires additional consideration in the clinical usage of PCSK9i, particularly with alirocumab. A positive-control sensitivity analysis was performed with atorvastatin, rosuvastatin, and ezetimibe as the reference groups. The results showed that, in comparisons among the 3 groups, the signal for alirocumab remained positive at the SOC level; however, the signal for evolocumab did not persist when compared to rosuvastatin. This shows that the signal for alirocumab is stronger, but the signal for evolocumab is less specific; these findings may reflect the baseline features of the dyslipidemic group.

It is worth mentioning that more women than men experienced PCSK9i-related RTMD adverse effects. There were 9646 female patients (56.53%) and 6921 male patients (40.56%). This phenomenon could be attributable to 2 sources. First, the IMPROVE cohort study found that PCSK9i levels are considerably greater in women than in men.^[21]^ In the Netherlands, women accounted for 42% of all PCSK9i patients, with women reporting 1.23 times more adverse events than men.^[22]^ As a result, women may be more susceptible to harmful effects. Second, women may be more emotionally vulnerable after experiencing adverse events, making them more likely to report their condition to pharmacovigilance centers.^[23]^ The larger proportion of women in relevant spontaneous reports could be attributed to differences in the patient population composition, reporting behavior, or gender-related perceptions of adverse events and reporting tendencies; however, this study was unable to discriminate between these variables.

In this investigation, the median age of patients reporting RTMD adverse events related to PCSK9i was 68 years, implying that the majority of adverse events occurred in older persons. A study based on the FAERS database found that several adverse events associated with evolocumab and alirocumab were more common in people aged over 65.^[24]^ Possible explanations for this phenomenon include clinical indications and prescription patterns, the rising prevalence of hyperlipidemia among older adults,^[25]^ and the expanded clinical use of PCSK9 inhibitors. While elderly age is common among those reporting RTMDs, this phenomenon is most likely due to the age distribution of patients receiving lipid-lowering therapy, a higher burden of comorbidities, and concurrent medication use, rather than a directly observable increase in clinical risk at specific age groups. These age-related findings are valuable reference points for clinicians and provide a foundation for the safe use of PCSK9i in elderly patients, as well as more effective disease management. However, because this study used a spontaneous reporting approach, it is impossible to determine the exact occurrence of this illness among different age groups.

Our data suggest that the United States had the highest number of RTMD adverse events (n = 15,031, accounting for 88.09%), which could be attributed to its dominance in PCSK9 inhibitor prescriptions. The most PCSK9i-related RTMD adverse events were recorded in 2018 (n = 8156), with evolocumab (n = 7499) being the most common. However, the number of reports fell substantially in 2020 (n = 929) and 2021 (n = 696), then increased in 2022 (n = 1154). This temporal distribution pattern might be associated with the COVID-19 pandemic. We suggest that the pandemic may have diverted some healthcare resources between 2020 and 2021, resulting in less attention to adverse events in other areas; once healthcare operations returned to normal in 2022, the number of RTMD-related adverse event reports resumed an upward trend. Regarding reporting timing, this study found that the median time to report PCSK9i-related RTMD adverse events was 7.5 days (IQR = 0.5–77.5 days), with 63.55% of reports made within 30 days of therapy beginning. This shows that close monitoring for potential RTMD adverse events may be required during the early phases of PCSK9i therapy.

This study has the benefits of a real-world study, but it also has several limitations. First, spontaneous reporting methods are prone to issues such as incomplete, missing, or erroneous reports, which can bring several confounding factors and uncertainties into the research. For example, when evaluating TTO and WSP, only 9902 reports (58%, 9902/17,063) were included, implying that only half of the reports were considered. This may produce findings that are not totally objective or truthful. Second, using a proportional analysis method for data mining can only reveal the proportion of a specific type of adverse event report among all reports; it cannot establish a causal relationship between the drug and the adverse event, nor does it calculate the incidence rate of the adverse event. Third, considering the lack of research in this area, the adverse effects revealed in this study still require experimental validation. Association analyses using the FAERS database can only describe clinical characteristics and statistical significance; they cannot determine causality or quantify risk. This study’s conclusions deserve additional confirmation through large-scale clinical prospective studies and experimental research.

In a retrospective investigation, we examined the reporting frequency and clinical features of RTMD adverse events related to PCSK9i. The outcomes of this study provide preliminary evidence for potential RTMD adverse effects in PCSK9i treatment. They simply suggest that such events need more attention in PCSK9i-related reports, which may assist physicians in monitoring these adverse events and implementing early interventions; nevertheless, this study does not show a causal association between PCSK9i and RTMD adverse events.

## Author contributions

**Conceptualization:** Hongzhu Chen.

**Data curation:** Hongzhu Chen, Lijing Li.

**Formal analysis:** Hongzhu Chen.

**Methodology:** Lijing Li.

**Visualization:** Lijing Li.

**Writing – original draft:** Hongzhu Chen, Lijing Li.

**Writing – review & editing:** Lijing Li.


