# Supplementary material for: A retrospective study of disproportionality analysis signals between proprotein convertase subtilisin/kexin 9 inhibitor use and respiratory, thoracic, and mediastinal disorders
Source: Medicine (Baltimore). 2026 Aug 28;105(35):e50089. doi: 10.1097/MD.0000000000050089 (PMC13529137; doi:10.1097/MD.0000000000050089)
Supplement: Supplementary file 1 [file medi-105-e50089-s001.docx]

**Supplement Table1** Summary of algorithms applied for signal detection.

| Algorithms | Equation | Criteria |
| --- | --- | --- |
| ROR | ROR =(a/b)/(c/d)  95%CI=e^1n(ROR)±1.96(1/a+1/b+1/c+1/d)^0.5^ | Lower 95 % CI>1, N≥3 |
| BCPNN | IC=log_2_^a(a+b+c+d)/[(a +c)(a+ b)]^  IC_025_=e^1n(IC)-1.96(1/a+1/b+1/c+1/d)^0.5^ | IC_025_ >0 |

a: number of reports containing both the suspect drug and the suspect adverse drug reaction;

b:number of reports containing the suspect adverse drug reaction with other medications (except the drug of interest);

c: number of reports containing the suspect drug with other adverse drug reactions (except the event of interest);

d: number of reports containing other medications and other adverse drug reactions.

ROR, reporting odds ratio;

N, the number of co-occurrences;

BCPNN, Bayesian confidence propagation neural network;

IC, information component;

IC_025_, the lower limit of the 95 % two-sided CI of the IC.
